# Molecular action of NZ2114, a superior plectasin derivative

**DOI:** 10.1038/s44259-026-00196-6

**Published:** 2026-05-04

**Authors:** Maik G. N. Derks, Shehrazade Jekhmane, Sourav Maity, Vicky Charitou, Cornelis J. Slingerland, Benjamin Vermeer, Mick van der Weijde, Charalampos Ntallis, Nathaniel I. Martin, Wouter H. Roos, Eefjan Breukink, Markus Weingarth

**Affiliations:** 1 NMR Spectroscopy, Department of Chemistry, Utrecht University, Utrecht, The Netherlands; 2 Membrane Biochemistry and Biophysics, Department of Chemistry, Utrecht University, Utrecht, The Netherlands; 3 Moleculaire Biofysica, Zernike Instituut, Rijksuniversiteit Groningen, Groningen, The Netherlands; 4 Biological Chemistry Group, Institute of Biology Leiden, Leiden University, Leiden, The Netherlands

**Keywords:** Biochemistry, Biotechnology, Chemical biology, Chemistry, Drug discovery, Microbiology, Structural biology

## Abstract

NZ2114 is a triple-mutant of the natural peptide antibiotic plectasin that targets the peptidoglycan precursor molecule Lipid II using a supramolecular action, involving assembly of plectasin-Lipid II complexes in a large oligomeric structure, that is enhanced by calcium ions. Due to its superior potency against *Staphylococcus aureus* strains, NZ2114 was the candidate that was advanced to clinical trials, and it has become the standard-template for the development of improved plectasin derivatives. However, the molecular underpinning for NZ2114’s improved potency remains opaque, with biochemical data pointing to chemical modification of the Lipid II target in *Staphylococci* that would impair the target binding capacity of plectasin but not the one of NZ2114. Here, using an integrative structural biology approach based on solid-state NMR, high-speed atomic force microscopy, and affinity assays, we demonstrate that both NZ2114 and plectasin bind effectively to *Staphylococcal* Lipid II variants, which means that NZ2114’s greater potency against *S. aureus* does not result from a difference in target binding. Instead, we show that the three residue substitutions in NZ2114 change its N-terminal fold, markedly increasing its sensitivity to calcium ions, which results in a different supramolecular action on the membrane surface. Altogether, our study provides new insights for the design of superior drug candidates.

## Introduction

The rise of multidrug-resistant bacteria calls for the development of new antibiotics^1–5^. Plectasin is a natural peptide antibiotic produced by the fungus *Pseudoplectania nigrella*^6^. It adopts a compact ‘αβ defensin’ fold (40 residues) stabilized by cysteine residues, characteristic of a broad family of host defense peptides found in invertebrates. Plectasin kills bacteria by targeting the peptidoglycan precursor Lipid II^7^ with high affinity, which inhibits the cell wall synthesis and presumably also alters the organization of the plasma membrane^8,9^. Its strong activity against a large panel of Gram-positive pathogens, along with favorable drug-like characteristics such as low toxicity in animal studies, commercially viable expression levels and high stability^6,10^, make plectasin an interesting candidate for the development of antibiotics.

**Fig. 1:**
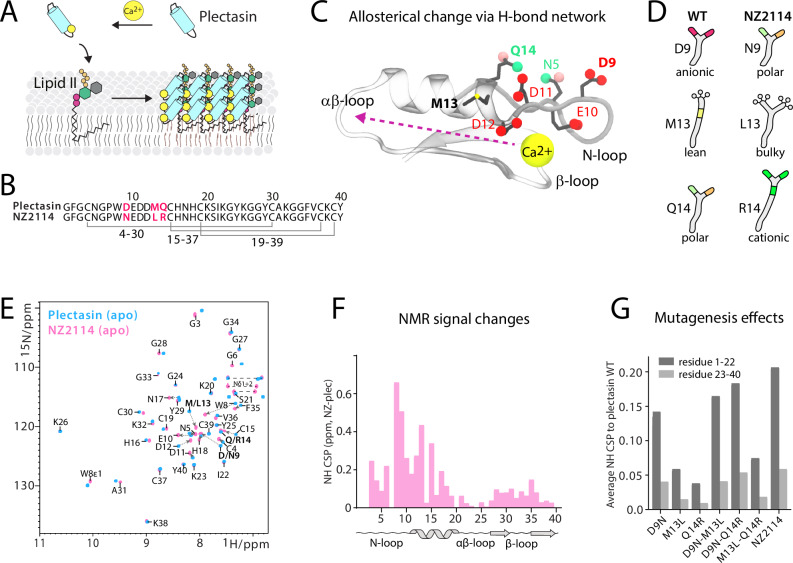
Triple-mutation in NZ2114 alters an extended hydrogen-bonding network. **A** Plectasin sequesters Lipid II in the bacterial plasma membrane using a Ca^2+^-dependent, supramolecular mechanism. **B** Amino acid sequence of plectasin and NZ2114. The three residue substitutions in NZ2114 are highlighted in red. **C** Orchestrated by a hydrogen bonding network and supramolecular contacts, Ca^2+^-binding to anionic residues in the N-loop changes the αβ-loop, which is involved in Lipid II-binding. **D** The three substitutions in NZ2114 that alter the hydrogen bonding network. **E** 2D NH solution NMR spectra of NZ2114 and plectasin. **F** Signal differences between NZ2114 and plectasin from NH solution NMR. **G** Average NH signal changes of NZ2114 and intermediary mutants compared to plectasin, split between the two halves of the peptides. Only amide backbone CSPs were used to calculate the average.

However, plectasin shows low efficacy against *Staphylococcus aureus*—including MRSA^6^—and *Enterococci* strains, a finding which incentivized the discovery of plectasin derivative NZ2114 by a high-throughput mutation and screening campaign^11^. NZ2114 is a triple mutant (D9N, M13L, Q14R) (Fig. 1B) with excellent efficacy against *staphylococci*, and *streptococci* in animal models of infection^11–13^, comparable to vancomycin or daptomycin, and this is the variant of plectasin that was eventually subjected to pre-clinical studies^14^. NZ2114 has subsequently been used as a template for a large number of drug development efforts, yielding attractive compounds with good activity against *Mycobacterium tuberculosis*^15^, MRSA^16^ and bacterial biofilms^17,18^, as well as improved drug characteristics such as proteolytic stability^19^, formulation stability^20^, and expression levels^21^.

Despite the popularity of NZ2114 for drug development, the molecular underpinning for its improved activity remains unclear, and it is also unclear why plectasin shows low activity against *S. aureus*. The only concrete scenario in the literature is that NZ2114 would bind with high affinity to chemically modified variants of Lipid II, such as the pentaglycine form that is found in *S. aureus* strains, while such modification would reduce the Lipid II-binding affinity of plectasin^22^. However, there is no direct evidence for this hypothesis.

Recently, we reported on the action of plectasin using integrated structural biology^9^. We showed that plectasin forms large supra-structures on the membrane to stably bind Lipid II (Fig. 1A). We have also demonstrated that such supramolecular mechanisms are commonly used by Lipid II-targeting antibiotics such as teixobactin^23^, clovibactin^24^, and many others^9^ (including the αβ-defensin copsin^25^). These supra-structures form quasi-permanently on relevant timescales, and hence increase the lifetimes of drug–target interactions^26,27^. What is special about plectasin is that bivalent cations (Ca^2+^, Mg^2+^) markedly modulate its supramolecular mechanism and antimicrobial potency^9^. We recently showed that bivalent cations coordinate to plectasin’s anionic patch (D9-E10-D11-D12). Cation binding then, orchestrated by an extended hydrogen bonding network and by interactions with adjacent peptides in the supra-structure, causes a change in the αβ-loop localized at the other end of plectasin, decisively enhancing the antibiotic’s Lipid II binding affinity and antimicrobial activity^9^ (Fig. 1C). Intriguingly, all the three residue-substitutions in NZ2114 are localized around the anionic patch, increasing the charge by +2, with likely implication for bivalent cation binding and the structural modulations governed by the hydrogen bonding network (Fig. 1C, D).

Here, using comprehensive structural biology and biophysical approaches, we compare the killing mechanism of the improved variant NZ2114 to plectasin. We demonstrate that NZ2114’s action is much more sensitive to the presence of bivalent ions, forming distinct supramolecular structures depending on the ion concentration. Moreover, we show that both NZ2114 and plectasin efficiently bind *staphylococcal* Lipid II variants, strongly suggesting that NZ2114’s superior potency against *S. aureus* cannot be attributed to differences in target binding, but rather points to the impact of different supramolecular actions.

## Results

### Mutations alter N-terminal loop

First, we sought to investigate if the three amino acid substitutions in NZ2114 change its structure compared to plectasin. To this end, we produced uniformly ^13^C,^15^N-labeled NZ2114 and plectasin peptides in *Escherichia coli* SHuffle® and carried out solution NMR studies. We note that purified NZ2114 indeed displayed 4-fold higher activity against MRSA than plectasin (Supplementary Table 1) in MIC assays, which is in line with previous studies^10^ that reported an 8 to 2-fold higher activity of NZ2114 against MRSA and MSSA strains.

Solution NMR data of NZ2114 and plectasin show that the peptides adopt the same overall fold (Fig. 1E), which is unsurprising as the global peptide’s structure is constrained by three disulfide bonds. However, we observed notable NH signal changes throughout the entire N-loop (G3-C15), as well as a prominent (>3 ^13^C ppm) signal change for K23 located in the αβ-loop (Fig. 1F and Supplementary Fig. 1A–G). As we recently showed, both N-loop and αβ-loop are critical for cation binding, target binding, and antimicrobial activity^9^. Considering the pronounced NMR signal changes in the N-terminus and given that two (D9N and Q14R) of the three substitutions in NZ2114 increase the total charge around the anionic patch, we wondered whether NZ2114 has altered bivalent cation-binding properties. Previously^9^, we showed by Isothermal titration calorimetry (ITC) that soluble plectasin binds bivalent cations (Ca^2+^ and Mg^2+^) with (sub-)µM affinity. Solution NMR, combined with paramagnetic Mn²⁺, further pinpointed the cation-binding site at the anionic patch, likely coordinated by aspartate and glutamate side chains. Additionally, ⁴³Ca solid-state NMR demonstrated that Ca²⁺ ions associate with plectasin upon its coordination with Lipid II in liposomes. We also observed that, in the Lipid II-bound state, the aspartate and glutamate side chains of plectasin rigidify in the presence of calcium ions, but not in their absence. Here, we show by ITC measurements that NZ2114 still coordinates Ca^2+^ and Mg^2+^ in solution in the (sub-)µM regime, which is like plectasin^9^ (Supplementary Fig. 2A, B). Next, to explore structural changes in the cation-binding mode, we conducted solution NMR experiments with paramagnetic Mn^2+^ ions, which quench signals of residues close to the ions (Supplementary Fig. 1H, I). These data indeed demonstrate differential ion binding for plectasin and NZ2114, with NZ2114’s N-terminal residues being less affected by paramagnetic ions. Altogether, these data strongly suggest a conformational change in the N-loop of NZ2114 relative to plectasin.

We aimed to untangle how the individual (D9N, Q14R, M13L) residue substitutions improve the potency of NZ2114. To this end, we recombinantly produced all six single and double substituted peptides (D9N, Q14R, M13L, D9N-M13L, D9N-Q14R, M13L-Q14R) and conducted activity assays (Supplementary Table 1). While the M13L substitution alone showed no effect, each of the D9N and Q14R single substitutions improved plectasin’s activity against MRSA, and the D9N, Q14R-double substitution displayed comparable activity to NZ2114. Next, we investigated the effect of the substitutions on plectasin’s conformational space by solution NMR (Fig. 1G, Supplementary Fig. 3). Strikingly, the D9N substitution strongly affects the fold of plectasin, causing allosteric structural changes in the entire N-terminus (G3-C15) and in the adjacent β-strand (Y29-V36). In contrast, we observed only minor signal changes for the M13L and the Q14R substitution. The overall signal changes are amplified in the D9N-Q14R double mutant. As both D9N and Q14R are hydrogen donors/acceptors, these data mean that the NMR signal changes observed for the triple mutant NZ2114 are based on changes in plectasin’s extended hydrogen bonding network, which connects the anionic patch with the opposing β-sheet and the αβ-loop. These conclusions are in line with MD simulations that, compared to plectasin, show increased flexibility of the N-loop in NZ2114 (Supplementary Fig. 4).

### Mutations increase calcium impact

**Fig. 2:**
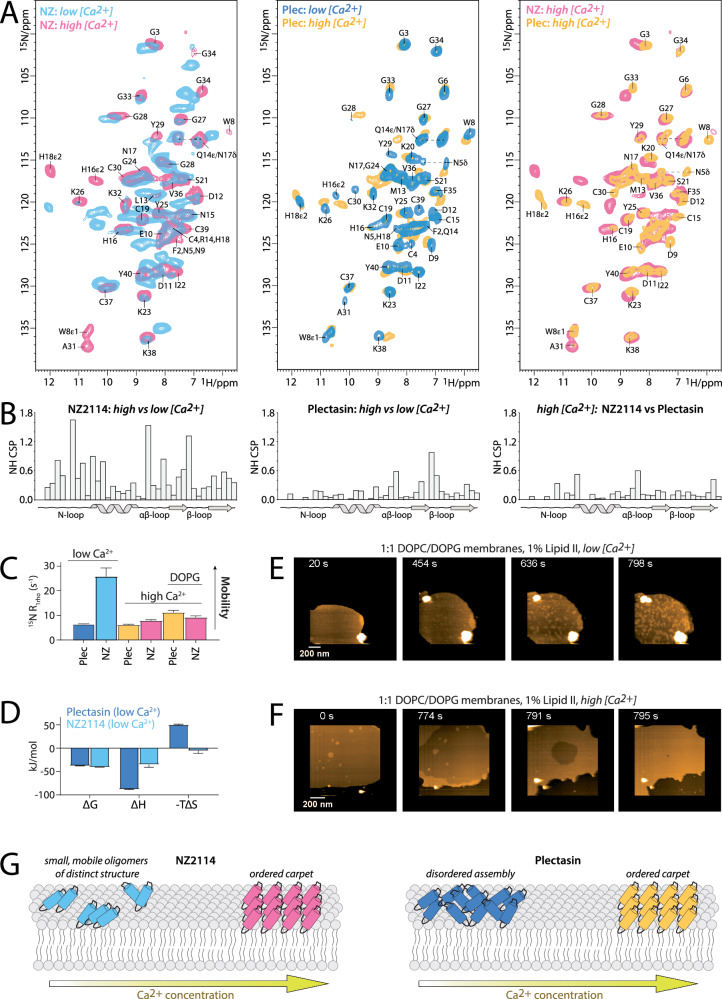
The supramolecular action of NZ2114 is highly sensitive to the Ca^2+^ concentration. **A** Superposition of 2D NH ssNMR spectra of Lipid-bound peptide-antibiotics acquired at different Ca^2+^ concentration in DOPC liposomes. (*Left panel*) NZ2114 at low (in light blue) and *high [Ca*^*2+*^*]* (red) concentration. (*Middle panel*) Plectasin at *low [Ca*^*2+*^*]* (dark blue) and *high [Ca*^*2+*^*]* (orange). (*Right Panel*) NZ2114 (red) and plectasin (orange) at *high [Ca*^*2+*^*]*. **B** NH signal differences (chemical shift perturbations [CSP]) of the spectra shown in (**A**). Signals were assigned using a combination of ^1^H-detected and ^13^C-detected 2D and 3D CC ssNMR experiments. **C** ssNMR ^15^N R_1rho_ relaxation probing microsecond dynamics of the peptide-antibiotics bound to Lipid II. The error bars show the standard error of the fit. **D** Thermodynamics parameters of Lipid II binding acquired by ITC, comparing plectasin (dark blue) and NZ2114 (light blue). The error bars show the standard deviation of titrations conducted in duplicate. **E** Snapshots of a time-lapse HS-AFM video (Supplementary Video 1) following the oligomerization of NZ2114 in supported DOPC/DOPG lipid bilayers doped with 1% lipid II. **F** Same as (**E**) but in the presence of 1 mM Ca^2+^ (Supplementary Video 2). **G** Comparative models of the of Ca^2+^-sensitive supramolecular binding modes of NZ2114 and plectasin on the plasma membrane. Note that Lipid II is not shown in the illustration.

Next, we investigated the Lipid II-bound state of NZ2114 in model membranes. To this end, we co-assembled ^13^C,^15^N-NZ2114 and Lipid II in liposomes and acquired solid-state NMR (ssNMR) data at 1 mM Ca^2+^ (‘*high [Ca*^*2+*^*]*’) or without supplementing additional Ca^2+^ to the buffer (‘*low [Ca*^*2+*^*]*’) (Fig. 2A, B [left panels]). Strikingly, we observed pronounced signal differences, with NZ2114 showing two clearly distinguishable states at *low [Ca*^*2+*^*]*, while only one of the two states is present at *high [Ca*^*2+*^*]* (Supplementary Fig. 5A). The Ca^2+^-sensitivity of NZ2114’s action is confirmed by the addition of EDTA at 1 mM Ca^2+^ concentration, which yields the *low [Ca*^*2+*^*]* ssNMR spectrum (Supplementary Fig. 5B), although with broadened signals that suggest a decrease in molecular order in the absence of Ca^2+^ ions. We henceforth denote the NZ2114-state that is only visible at *low [Ca*^*2+*^*]* as the ‘*low [Ca*^*2+*^*] state’*, and conversely, the other state as the ‘*high [Ca*^*2+*^*] state’*. These two states show notable structural differences throughout the entire NZ2114-peptide, especially in the N-loop, the αβ-loop, and the β-loop (Supplementary Fig. 6). The largest signal change was observed for αβ-loop residue Y29, a residue that is likely in proximity of the Lipid II sugars^9^, which showed a massive so-called secondary chemical shift (SCS) difference of 6 ^13^C-ppm at *high [Ca*^*2+*^*]*. We interpret these two states as distinct supramolecular structures of Lipid II-bound NZ2114 that form in response to the Ca^2+^ concentration of the environment. In contrast, Lipid II-bound plectasin is markedly less affected by the Ca^2+^ concentration (Fig. 2A, B [middle panels]). Plectasin adopts only one state of similar conformation at both *low [Ca*^*2+*^*] and high [Ca*^*2+*^*]* concentrations, with only small local conformational differences around the anionic patch and the αβ-loop. Remarkably, the ‘*high [Ca*^*2+*^*] states’* of Lipid-II-bound NZ2114 and plectasin resemble each other strongly, with small local differences in the N-loop, the αβ-loop, as well as a small destabilization of the β-sheet in NZ2114 (Fig. 2A, B [right panels] and Supplementary Fig. 6).

We next analyzed the impact of the calcium concentration on the conformational dynamics of the Lipid II-bound antibiotics. To this end, we measured ssNMR ^15^N R_1rho_ relaxation rates that are sensitive to µs timescale dynamics (Fig. 2C)^28,29^. Strikingly, at *low [Ca*^*2+*^*]*, Lipid II-bound NZ2114 shows much higher dynamics than plectasin, while the dynamics are similar at *high [Ca*^*2+*^*]*. These data corroborate that NZ2114 is substantially more sensitive to Ca^2+^ concentration than plectasin. We wondered what the markedly increased dynamics of NZ2114 at *low [Ca*^*2+*^*]* mean for its Lipid II binding capacity. To this end, we dissect the Lipid II-binding energetics of NZ2114 and plectasin at *low [Ca*^*2+*^*]* using ITC (Fig. 2D and Supplementary Fig. 2C). The measurements show similar Lipid II binding affinities for NZ2114 (Kd = 147 ± 7 nM) and plectasin (Kd = 423 ± 25 nM). However, while plectasin pays a high entropic penalty (-TΔS = 50.1 ± 1.3 kJ/mol) upon binding to Lipid II, NZ2114 does not (-TΔS = 5.4 ± 5.6 kJ/mol), in line with a greater mobility—and thus higher entropy—of the bound state^30^. These data clearly demonstrate that NZ2114 and plectasin employ different molecular actions and different sensitivities to the calcium concentration. It is important to highlight that ITC captures the cumulative thermodynamic contributions of the binding event. Given that target binding and oligomerization are intrinsically linked for plectasin and NZ2114, ITC thus reflects the ‘effective affinity’ of the interaction.

Next, we probed the effect of calcium on the formation of supra-structures on the membrane surface. Recently, using high-speed atomic force microscopy (HS-AFM)^31,32^, we showed that plectasin captures Lipid II by forming large supra-structures on the membrane surface^9^. These massive plectasin supra-structures form in the absence of calcium, but the molecular order of the arrangement is substantially increased in the presence of calcium. Strikingly, HS-AFM data reveal a notably different effect of calcium on the supramolecular mechanism of NZ2114 (Fig. 2E, F and Supplementary Fig. 7. See also Supplementary Fig. 8 for a direct comparison of HS-AFM data of NZ2114 and plectasin). In the absence of Ca^2+^, NZ2114 forms highly dynamic, smaller oligomers to target Lipid II. In the presence of Ca^2+^, the observed NZ2114 – Lipid II supra-structures are indistinguishable from the previously observed^9^ plectasin – Lipid II supra-structures. These HS-AFM data are in excellent agreement with ssNMR. Both techniques clearly demonstrate that calcium ions have a much larger impact on the action of NZ2114 than on plectasin’s action, and both techniques show that Lipid II-bound NZ2114 adopts a more mobile state at *low [Ca*^*2+*^*]*.

Together, these data consistently demonstrate that NZ2114 and plectasin deploy distinct supramolecular Lipid II-targeting mechanisms (Fig. 2G). At *low [Ca*^*2+*^*]*, plectasin assembles into a large but disordered supramolecular carpet on the membrane surface, which transitions into a highly ordered supra-structure at *high [Ca*^*2+*^*]*. In contrast, NZ2114 adopts a unique structure that organizes in small and mobile oligomers at *low [Ca*^*2+*^*]*. At *high [Ca*^*2+*^*]*, NZ2114 undergoes a global conformational change and converts into ordered supra-structures that closely resemble those of plectasin.

### Lipid II variants are efficiently targeted

Previous reports^22^, based on indirect biochemical evidence, suggested that plectasin’s low activity against *S. aureus* would be based on its reduced capacity to bind special Lipid II variants of *S. aureus*, while NZ2114 would be able to target such variants more efficiently. These reports prompted us to gauge if the superior activity of NZ2114 could be due to a distinct Lipid II binding interface. To this end, we first assembled liposomes with ^13^C-labeled Lipid II and (unlabeled) ^12^C-peptides and conducted ssNMR studies. However, we obtained similar NMR signals for Lipid II bound to either plectasin or NZ2114, suggesting similar Lipid II binding interfaces (Supplementary Fig. 9).

**Fig. 3:**
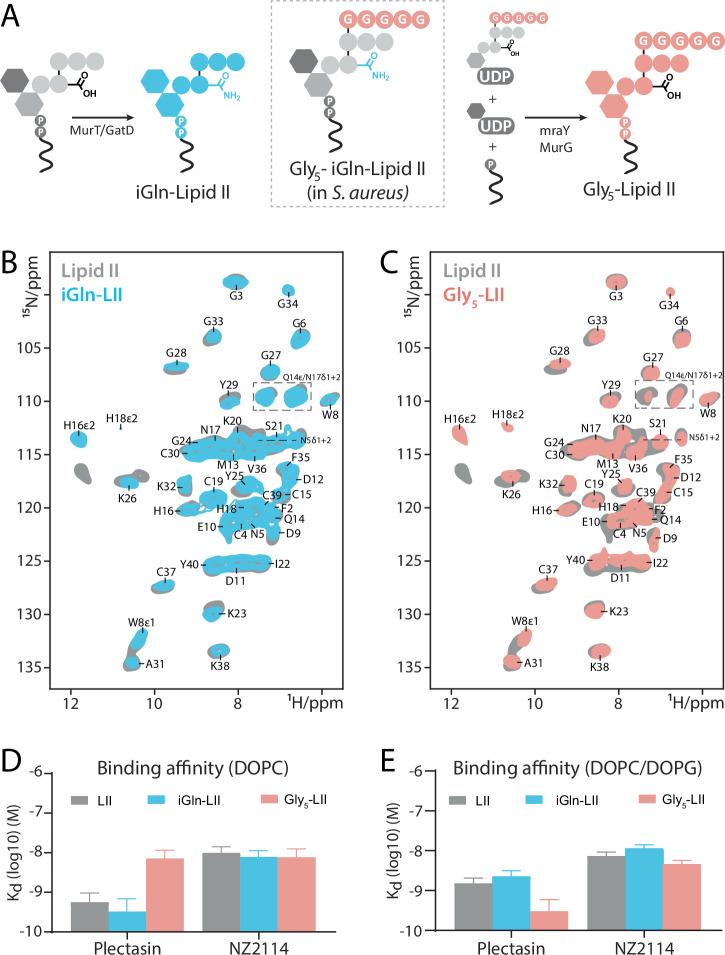
Efficient binding to *Staphylococcal* Lipid II variants. **A** Schematic illustration of the biosynthesis and structure of amidated Lipid II (iGln-LII), pentaglycine Lipid II (Gly_5_-LII), and amidated pentaglycine Lipid II (iGln-Gly_5_-LII), with the latter being present in *S. aureus*. **B**, **C** 2D NH ssNMR spectra comparing plectasin in complex with three Lipid II variants. Samples were measured at *high [Ca*^*2+*^*]* and in anionic membranes (1:1 molar ratio of DOPC/DOPG). Note that the large apparent shifts of the histidine sidechains in the ^15^N-dimension are due to differences in spectral folding. **D**, **E** Comparison of the binding affinities of plectasin and NZ2114 to the Lipid II variants in zwitterionic (**D**) or anionic (**E**) membranes at *high [Ca*^*2+*^*]*, obtained using intrinsic tryptophan fluorescence. The error bars show standard error of the fit. Titrations were conducted in duplicate to ensure replicability.

Next, we dissected if plectasin’s activity is indeed compromised by modifications of its target - Lipid II. To this end, we used ssNMR to directly investigate plectasin’s interaction with two prevalent *staphylococcal* Lipid II variants: i) Amidated Lipid II (iGln-LII), in which pentapeptide-residue γ-Glutamate-2 is converted to γ-isoglutamine; and ii) Pentaglycine Lipid II (Gly_5_-LII), in which a pentaglycine unit is attached to Lysine-3 of the pentapeptide. We then collected 2D NH ssNMR spectra of plectasin in complex with the three Lipid II variants in anionic liposomes and at *high [Ca*^*2+*^*]* (Fig. 3B, C). Strikingly, the ssNMR spectra are nearly identical for all target variants. These data clearly demonstrate that plectasin can efficiently bind to *staphylococcal* Lipid II and that Lipid II modifications do not affect the conformation of the bound state of plectasin.

That Lipid II modifications do not prevent plectasin from interacting with its target is also demonstrated by comprehensive binding affinity measurements, based on intrinsic tryptophan fluorescence, that we conducted at *high [Ca*^*2+*^*]* and in zwitterionic as well as anionic membranes. These data confirm (Fig. 3D, E) that the modifications only have a small effect on the Lipid II binding affinity, demonstrating that both NZ2114 and plectasin can bind Lipid II and its *staphylococcal* variants with high affinities (*K*_*d*_ = 10^-8^ to 10^-9 ^M). Most notably, in both zwitterionic and anionic membranes, plectasin binds Lipid II variants with higher affinity than NZ2114.

Finally, as NZ2114 has increased overall charge (+2) compared to plectasin, we thought to probe the role of the overall charge of the cell envelope for the differential activities of the two peptides. To this end, we tested the activity of plectasin and NZ2114 against a *Bacillus subtilis* DltB knockout, which causes a global loss of teichoic acid D-alanylation and concurrently a more negatively charged cell envelope (Supplementary Fig. 10). Plectasin and NZ2114 displayed similarly increased activity against the DltB mutant, strongly suggesting that the cell surface charge is not a discriminatory factor between NZ2114 and plectasin.

Considering these affinity data and the high similarity of the binding interfaces observed by ssNMR, it seems highly unlikely that modifications of Lipid II are the cause for NZ2114’s improved potency against *S. aureus*.

## Discussion

NZ2114 is a triple mutant derivative of the natural antibiotic plectasin^6^ that exhibits significantly enhanced activity against *Staphylococcus aureus* strains compared to the original peptide.

Our data disclose that, depending on the concentration of Ca^2+^ in the environment, the triple-mutant NZ2114 uses a markedly different supramolecular action compared to plectasin. We show that the three residue substitutions in NZ2114 alter its N-terminal fold and its hydrogen-bonding network connecting the Ca^2+^-binding site to the αβ-loop, which is critical for Lipid II targeting. These conformational changes increase NZ2114’s sensitivity to Ca^2+^ ions, adopting different molecular structures and employing different supramolecular Lipid II targeting mechanisms at *low* and *high [Ca*^*2+*^*]* concentrations. At *low [Ca*^*2+*^*]*, ssNMR and HS-AFM consistently show that NZ2114 binds Lipid II in dynamic, small oligomeric assemblies, while a massive and rigid supra-structure is formed in the presence of *high [Ca*^*2+*^*]*. This behavior clearly contrasts from plectasin, on which Ca^2+^-binding has more subtle effects^9^: Plectasin binds Lipid II in large carpet-like assemblies at both *low* and *high [Ca*^*2+*^*]*, but the structural order of the assemblies increases as the Ca^2+^ concentration rises. Moreover, while NZ2114 binds Lipid II in two different molecular conformations depending on the Ca^2+^ concentration, Ca2+-dependent structural changes in plectasin are more subtle and local. Remarkably, despite their different Ca^2+^-sensitivities, their supra-structures are similar at *high [Ca*^*2+*^*]*, with both peptides forming dense carpet-like assemblies on the membrane surface in which Lipid II gets efficiently sequestered.

Importantly, using comprehensive structural and binding affinity data, we show that both plectasin and NZ2114 efficiently bind to Lipid II variants common in *S. aureus*. Thereby, our findings challenge previous reports that proposed that the low activity of plectasin against *S. aureus* was due to the amidation of Lipid II^22^. This hypothesis was based on the observation that plectasin displayed increased activity against an amidation-depleted strain of *S. aureus*. The molecular rationale for the increased activity was that plectasin could use its His18 side chain to form a salt bridge^8^ with the D-γ-glutamic acid of Lipid II-pentapeptide. In our previous paper, we argued that this salt bridge is a modeling bridge^8^, and in this study, we indeed explicitly refute this hypothesis, showing that the conversion of D-γ-glutamic acid to a D-γ-isoglutamine does not reduce plectasin’s binding affinity. In hindsight, this seemed an unlikely hypothesis, as many Gram-positive bacteria contain amidated Lipid II, including species such as *Streptococcus pneumoniae*^33,34^ against which wild-type plectasin displays particularly high activity^6^. Likely, depletion of Lipid II amidation has a general weakening effect on the cell envelope, which is expected to act synergistically with plectasin action. An alternative explanation is that a global increase in negative charge in the cell envelope increases plectasin activity, but in the context of our DltB knockout activity data (Supplementary Fig. 10), this is presumably not specific either. Moreover, the observation that binding of NZ2114 and plectasin to Lipid II was largely unaffected by both amidation and the presence of the pentaglycine unit (the bridging peptide is hyper-variable between bacterial species^35^) suggests that NZ2114 and plectasin are also capable of binding to Lipid II variants with other modifications at these sites. Therefore, the development of resistance by modification of these portions of the pentapeptide of Lipid II appears unlikely. This is in line with the observation that plectasin did not display cross-resistance with penicillin in *S. pneumoniae*^6^, as the enrichment of L-Ser/L-Ala or L-Ala/L-Ala as the bridging peptide is required to enable penicillin resistance by modification of PBPs^36,37^, and the finding that the related defensin actifensin could efficiently target D-Ala-D-Lac-Lipid II (ref. 38).

Although we do not know the precise molecular reason why plectasin is less active against *S. aureus*, our data indicate that alterations in the direct target-binding of these peptides to Lipid II are not the principal cause. Rather, our data point to the impact of different supramolecular actions for NZ2114’s superior activity, although we stress that the causal relevance of such differences for the observed variation in killing efficacy between plectasin and NZ2114 remains speculative. Given our previous findings^9^ that both plectasin and the αβ-defensin copsin^25^ employ supra-structures to bind Lipid II, it appears probable that this mechanism is conserved among Lipid II-targeting defensins. In this regard, mechanistic studies of oyster defensin Cg-Def^39^ also hints of the formation of a supramolecular assembly. Cg-Def, upon Lipid II binding, displayed a remarkably low dissociation rate constant *k*_off_, characteristic of supramolecular assemblies, and binding of Lipid II to Cg-Def resulted in the formation of an insoluble complex, in line with the formation of larger supramolecular aggregates. Intriguingly, contrary to the presumably more common supramolecular mechanism of Lipid II binding defensins, the Ca²⁺-sensitivity is likely most pronounced in plectasin and its derivatives, while other αβ-defensins that have been experimentally shown to interact with Lipid II have either fewer (for example, orzyeansin or eurocin^40^) or no anionic residues (for example, copsin^25^) in the N-loop (Supplementary Fig. 11). In general, significant gaps remain in our molecular understanding of how the supramolecular mechanisms of Lipid II-binding defensins are mediated^38^.

## Methods

### Plectasin and variants production

All plasmids were obtained by site-directed mutagenesis starting from the plectasin-pET SUMO plasmid as described in the QuikChange II site-directed mutagenesis kit manual (Agilent) using KOD Hot Start DNA Polymerase (Novagen). After thermocycling and digestion with DpnI, the plasmids were transformed into *E. coli* DH5α, single colonies were selected, and plasmid DNA was isolated. All isolated plasmids were sequenced to verify the correct insertion of the desired mutation(s). Plectasin and variants were produced in SHuffle® *E. coli* essentially as previously described^9^. In short, SHuffle cells carrying 6xHis-SUMO-plectasin plasmids (variants) were grown in M9 medium and expression was induced when the OD_600_ reached 0.6 by 0.5 mM IPTG and incubated further at 37 °C for 4 h. For some mutants, overexpression was done overnight at 30 °C, with varying success, when cultures were particularly slow growing. Cells were subsequently harvested, resuspended in 50 mM NaPi, 150 mM NaCl, 25 mM imidazole, 1 mM β-mercaptoethanol, pH = 8.0, and lysed by probe sonication in the presence of lysozyme and benzonase. After removal of cell debris by centrifugation, the lysate was filtered and purified on Ni-NTA resin, using a single-step elution with 400 mM imidazole in the same buffer. SUMO was cleaved with Ulp1 protease, and final purification was done on a Superdex 30 Hiload 26/60 column. Combined pure fractions were stored at -20 °C and concentrated using 3.5 kDa cutoff Amicon Ultra filters just before use. Note that plectasin (and variants) display low colloidal stability at neutral pH above ~0.3–0.5 mM (particularly during concentration peptide using ultrafiltration), forming precipitates. If required, precipitated protein can be resolubilized under acidic conditions (pH < 3) and subsequently repurified.

### Lipid II synthesis

Lipid II was synthesized and purified as previously described^9,41^. iGln-LII was produced enzymatically from Lipid II using the MurT/GatD enzyme complex^34^, while Gly_5_-LII was synthesized from undecaprenol and Park’s nucleotide pre-modified with the pentaglycine on Lys3 (produced by semi-synthesis from UDP-MurNAc^42^) (Fig. 3A, Supplementary Fig. 12). In detail, iGln-LII was synthesized by enzymatic conversion of Lipid II with MurT-GatD in the presence of 0.5 w/v% Triton-X100, 5 mM MgCl_2_, 6 mM ATP, 7.5 mM glutamine in 100 mM Tris at pH = 8.0 and purified using DEAE-cellulose after extraction with BuOH/6 M pyridine-acetate pH = 4.2. In several synthesis batches, some cardiolipin co-eluted with iGln-LII. In these cases, an additional purification step was required. Combined fractions were loaded on a silica 60 column equilibrated in chloroform and eluted using a gradient of methanol in chloroform. The MurT-GatD enzyme complex was produced in *E. coli* BL21 Star^TM^ (DE3) using a modified pET-30 plasmid as previously described^34^. After expression, harvested cells were resuspended in 100 mM Tris (pH = 8.0) to ~OD_600_ = 100 and treated with lysozyme, DNAse I, and RNAse A for 15 min at room temperature. Cells were subsequently lysed by probe sonication and cell debris was removed by centrifugation (38k x g, 4 °C, 15 min). The supernatant was flash-frozen in aliquots and stored at -80 °C until use without any purification. Gly_5_-Lipid II was synthesized analogously to unmodified Lipid II using UDP-MurNAcPP-Gly_5_ as a substrate (obtained as a kind gift from Matthieu Fonvielle, semi-synthesis described in ref. ^42^.). Synthesis was optimized for optimal conversion relative to the modified Park’s nucleotide, using a 5-fold excess of undecaprenyl phosphate and a 20-fold excess of UDP-GlcNAc. Reactions were monitored using TLC (0.2 mm silica gel 60, CHCl_3_/MeOH/H2O/25% aq NH_3_ 88:48:10:1 v/v/v/v) and stopped when no more Gly_5_-Lipid I (evident as a second spot running slightly higher) could be detected. The mraY^43^ and MurG^44^ enzymes used in these reactions were overexpressed in *E. coli* as previously described, and after harvesting the cells were washed once with 100 mM Tris (pH = 8.0), resuspended in the same buffer and immediately flash-frozen in aliquots (at OD_600_ = ~ 100-200). Thawed cell suspensions were added to the reaction mixtures and briefly sonicated to initiate synthesis (diluted in reaction mixture to approximately OD_600_ = 2-4).

### Tryptophan fluorescence spectroscopy

Tryptophan fluorescence experiments were conducted on a Cary Eclipse (FL0904M005) fluorimeter under constant stirring in a 4 × 10 mm quartz cuvette oriented perpendicular to the excitation beam at 20 °C. Emission spectra were recorded in 50 mM HEPES, 150 mM NaCl at pH = 7.2 and 20 °C for NZ2114 and plectasin titrated with 2 mol% Lipid II in DOPC or a 1:1 molar ratio of DOPC/DOPG (200 nm LUVs). Fluorescence was excited at 280 nm (5 nm slit) and recorded in 1 nm intervals between 300 and 400 nm using 1 second of averaging per point. Spectra were corrected for a blank with just buffer and for scattering. Scattering corrections were calculated from a separate titration, omitting the peptide in the cuvette. Corrected spectra were fit with log-normal functions, and the wavelength of maximum fluorescence was fit as a function of Lipid II concentration, using a quadratic binding equation assuming 1:1 stoichiometry. All titrations were conducted in duplicate. For affinity measurements of NZ2114 in the presence of EDTA we used a peptide concentration of 1 µM, which minimizes the contribution of scattering and reduces noise compared to the lower 100 nM peptide concentration that is necessary to obtain meaningful fits of titrations in the presence of 1 mM CaCl_2_.

### Isothermal titration calorimetry (ITC)

ITC measurements were conducted using a low-volume Affinity ITC (TA instruments) at 37 °C and under constant stirring at 125 rpm. Samples were degassed for 10 min before starting the experiments. The cell was filled with plectasin (-variant) in 50 mM HEPES, 150 mM NaCl at pH = 7.2 and titrated with large unilamellar vesicles (LUVs) or bivalent cation in the same buffer. 2% Lipid II in DOPC LUVs were made by the extrusion technique, using 200 nm filters. Titrations were performed in duplicates, and integrated heats were fit using an independent binding model in the NanoAnalyze software.

### MIC assays

Plectasin and variants were serially diluted in cation-adjusted Mueller-Hinton broth (CAMHB) in round-bottom polypropylene 96-well plates (50 µL per well). Bacterial strains (*Staphylococcus simulans 22, Staphylococcus aureus USA300* or *Staphylococcus aureus ATCC29213*) were revived from glycerol stocks on blood agar plates. Individual colonies were inoculated in tryptic soy broth and incubated at 37 °C under shaking until the OD_600_ reached 0.5. Suspensions were diluted 100 times in CAMHB and mixed with the peptide dilutions (50 µL per well). Plates were covered by a gas-permeable adhesive membrane and incubated at 37 °C while shaking. Minimum inhibitory concentrations (MICs) were determined by visual inspection after 16-20 hours of incubation. MIC assays were conducted in triplicate.

### Antibiotic susceptibility agarose diffusion assays

400 µL of *Bacillus subtilis* B168 or *B. subtilis* B168 ΔDltB (both obtained as kind gift from Richard Daniel) overnight TSB cultures were gently mixed into 40 mL hand-warm molten TSB containing 1.8 w/v% agarose and poured into 12×12 cm square petri dishes and left to solidify. Plectasin and NZ2114 were serially diluted in PBS and 10 µL of each concentration was spotted on the solidified plates and left to dry. Next, the plates were stored at 4 °C for 2 h and then incubated overnight (18–20 h) at 30 °C and subsequently imaged. Experiments were conducted in biological duplicate.

### ssNMR sample preparation

All ssNMR samples were made by hydration of lipid films containing 4 mol% Lipid II with plectasin or NZ2114 in buffer. For samples without calcium we used 50 mM NaPi, 150 mM NaCl at pH = 7.2 (with the addition of 0.5 mM EDTA when indicated), while for experiments in the presence of 1 mM CaCl_2_, we used HEPES instead. Appropriate lipid stocks in chloroform or 2:1 chloroform-methanol were mixed in glass tubes, dried under N_2_ in hand-warm water, and exposed to high vacuum for 20 minutes to remove residual organic solvent. Lipid films were hydrated with peptide solutions by extensive vortexing and then compacted using ultracentrifugation (100,000–150,000 x g, for 30–60 min at 4 °C) and then transferred to ssNMR rotors assisted by centrifugation.

### NMR spectroscopy

Solution NMR spectra were recorded at 14 T (600 MHz ^1^H frequency) and a sample temperature of 298 K. Paramagnetic relaxation enhancement experiments were conducted in the presence of 1 mM MnCl_2_. 3D backbone walk experiments and PRE of NZ2114 were conducted on 120 µM ^13^C^15^N-NZ2114 in 50 mM NaPi, 150 mM NaCl, 5 v% D_2_O, pH = 7.0 at 14.1 T (600 MHz ^1^H frequency) and 298 K sample temperature. All other solution NMR experiments were conducted at 20–50 µM peptide in 50 mM NaPi, 150 mM NaCl, 10 v% D_2_O, pH = 7.2.

^1^H-detected ssNMR experiments were conducted at 57-60 kHz MAS, a magnetic field strength of 16.4 T (700 MHz ^1^H frequency), and a sample temperature of approximately 305 K. Low-power PISSARRO^45^ (^1^H decoupling amplitude of 0.25 *ν_r_) was applied for decoupling. ^15^N-T_1rho_ relaxation experiments were conducted at 60 kHz MAS using an 18 kHz spin-lock field on ^15^N. 2D ^13^C-^13^C spectra were recorded at 16.4 or 22.3 T (700 and 950 MHz ^1^H frequency) using PARISxy^46^ recoupling (recoupling amplitude at ~0.75 * ν_r_) and PISSARRO or SPINAL64^47^ high power decoupling at 42 or 15–17 kHz MAS. Chemical shift assignments were obtained using ^1^H-detected 3D CαNH, Cα(CO)NH and CONH experiments as previously described^48^.

SCSs were calculated from Cα and Cβ chemical shifts as previously described^49^. Both SCSs and CO CSPs between plectasin and NZ2114 were corrected for the difference in random coil chemical shift of the mutated residues.

Chemical shift assignments of NZ2114 backbone in solution or bound to Lipid II in DOPC in the presence of calcium are shown in Supplementary Tables 2 and 3, respectively.

### Molecular Dynamics Simulations

Molecular dynamics simulations of the three plectasin variants in solution were performed using NAMD (version 3.0b6)^50^ with the CHARMM36m force field^51^. Input structures were prepared using the experimental structures with PDB IDs 3e7u (ref. ^52^) (wild type) and 6k50 (ref. ^53^) (NZ2114). Systems were solvated with TIP3P water and neutralized with 150 mM KCl. Production runs of 100 ns in the NPT ensemble were performed with a 2 fs time step at 310 K (Langevin dynamics method with a coupling coefficient of 1 ps^−1^) and 1 atm (Nosé-Hoover Langevin piston method with a piston period of 50 fs and a piston decay time of 25 fs). Nonbonded interactions were truncated at 12 Å with a 10 Å switching function, and the Particle Mesh Ewald method handled long-range electrostatics. Three independent replicas were conducted for each variant. Data were collected every 100 ps and subsequent trajectory analysis was done with the Visual Molecular Dynamics (VMD) software (version 1.9.4a57) (ref. ^54^).

### High-Speed Atomic Force Microscopy

All HS-AFM experiments were performed on a supported lipid bilayer (SLB) composed of DOPC and DOPG at equal molarity and 1% Lipid II on a mica substrate. The SLB was formed by absorption of LUVs onto freshly cleaved mica. Two microliters of diluted LUVs were incubated on a freshly cleaved, 1.5 mm diameter mica disc attached to a small glass rod (HS-AFM sample stage). After incubation for 5 to 10 min, the surface was washed 3 to 5 times with PBS. The localization of SLB patches was confirmed by HS-AFM imaging in PBS. Once an SLB patch was localized, a concentrated NZ2114 (in PBS) solution (60 µl) was added to the AFM recording chamber to achieve a final NZ2114 concentration of 1 µM. The experiments were repeated in the presence and absence of 1 mM CaCl_2_. All experiments were reproduced more than 3 times each. All HS-AFM data were taken in amplitude modulation mode using a sample scanning HS-AFM [Research Institute of Biomolecule Metrology (RIBM), Japan]. Short cantilevers (USC-F1.2-k0.15, NanoWorld, Switzerland) with a spring constant of 0.15 N/m and a resonance frequency around 0.6 MHz were used. The cantilever-free amplitude is 2 nm, and the set-point amplitude for the cantilever oscillation was set around 1.6 nm. Images were processed using ImageJ.

## Supplementary information

Derks_et_al_Supporting_information_2nd_revision

Supportin_Video1.

Supportin_Video2.

## Data Availability

Experimental solid-state NMR raw data are available via Zenodo at [10.5281/zenodo.18314779].
