# Supplementary material for: Molecular action of NZ2114, a superior plectasin derivative
Source: NPJ Antimicrob Resist. 2026 May 4;4:34. doi: 10.1038/s44259-026-00196-6 (PMC13139365; doi:10.1038/s44259-026-00196-6)
Supplement: Supplementary file 1 — Derks_et_al_Supporting_information_2nd_revision [file 44259_2026_196_MOESM1_ESM.pdf]

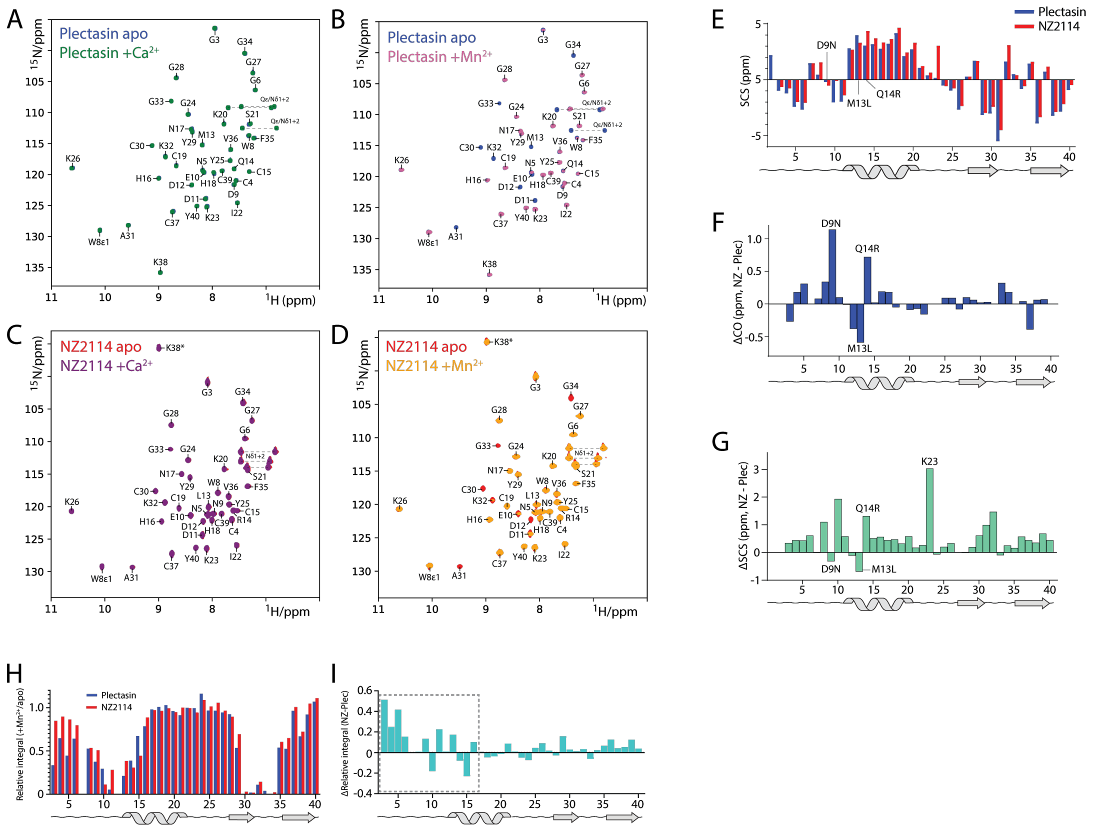

Supplementary Figure 1. (A) 2D $^{15}N^{1}H$-HSQC spectra of 42 µM plectasin before and after addition of 1 mM Ca^{2+} (at *high [Ca^{2+}])*. (B) Same but with 2 mM Mn^{2+}, which causes paramagnetic relaxation enhancement. (C) and (D) Same as A) and B) but with 120 µM NZ2114. *K38 resonance is aliased in the ^{15}N-dimension from ~135 ppm. (E) Absolute Cαβ secondary chemical shifts (SCS) for plectasin and NZ2114 in solution. (F) CO CSPs between NZ2114 and plectasin in solution. (G) Difference in Cαβ SCS between NZ2114 and plectasin. (H) Relative integrals (normalized) from B) and D) as a function of residue number. (I) Difference in relative integral from H) between the two peptides, showing differences in paramagnetic relaxation enhancement due to Mn^{2+}. Diagram shows the relative differences between the integral ratios of NH signals before and after addition of 2 mM Mn^{2+} of plectasin and NZ2114.

A

Ca²⁺ titration to NZ2114 (40 µM)

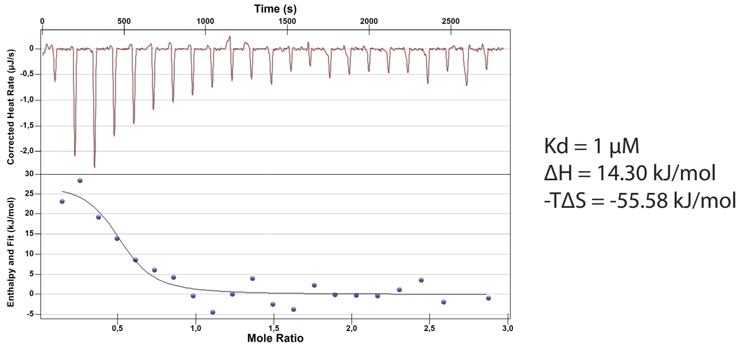

B

Mg²⁺ titration to NZ2114 (40 µM)

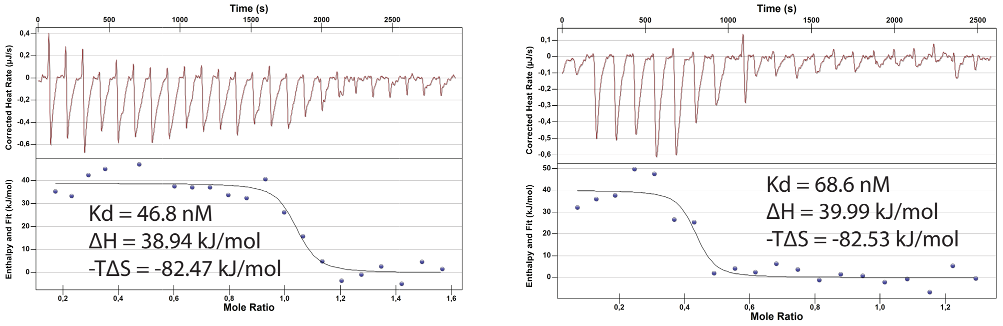

C

2% Lipid II in DOPC titrations

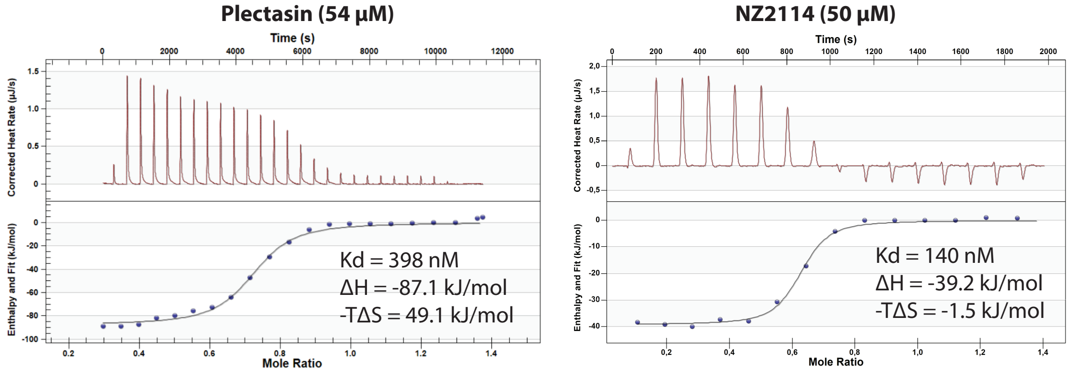

D

Exp	Plectasin #1	Plectasin #2	AVG	SD	NZ2114 #1	NZ2114 #2	AVG	SD
Kd (nM)	398	447	422.5	24.5	140	153	146.5	6.5
ΔG (kJ/mol)	-38.0	-37.7	-37.8	0.2	-40.7	-40.5	-40.6	0.1
ΔH (kJ/mol)	-87.1	-88.7	-87.9	1.1	-39.2	-31.1	-35.1	5.7
-TΔS (kJ/mol)	49.1	51.0	50.1	1.3	-1.5	-9.4	-5.4	5.6

Supplementary Figure 2. (A) & (B) ITC studies of the binding of soluble NZ2114 to Ca^{2+} and Mg^{2+}. (C) ITC studies of the binding of plectasin and NZ2114 to Lipid II in DOPC membranes at *low [Ca^{2+}]* conditions. (D) a table containing thermodynamic fit parameters for duplicates of the experiments shown in C.

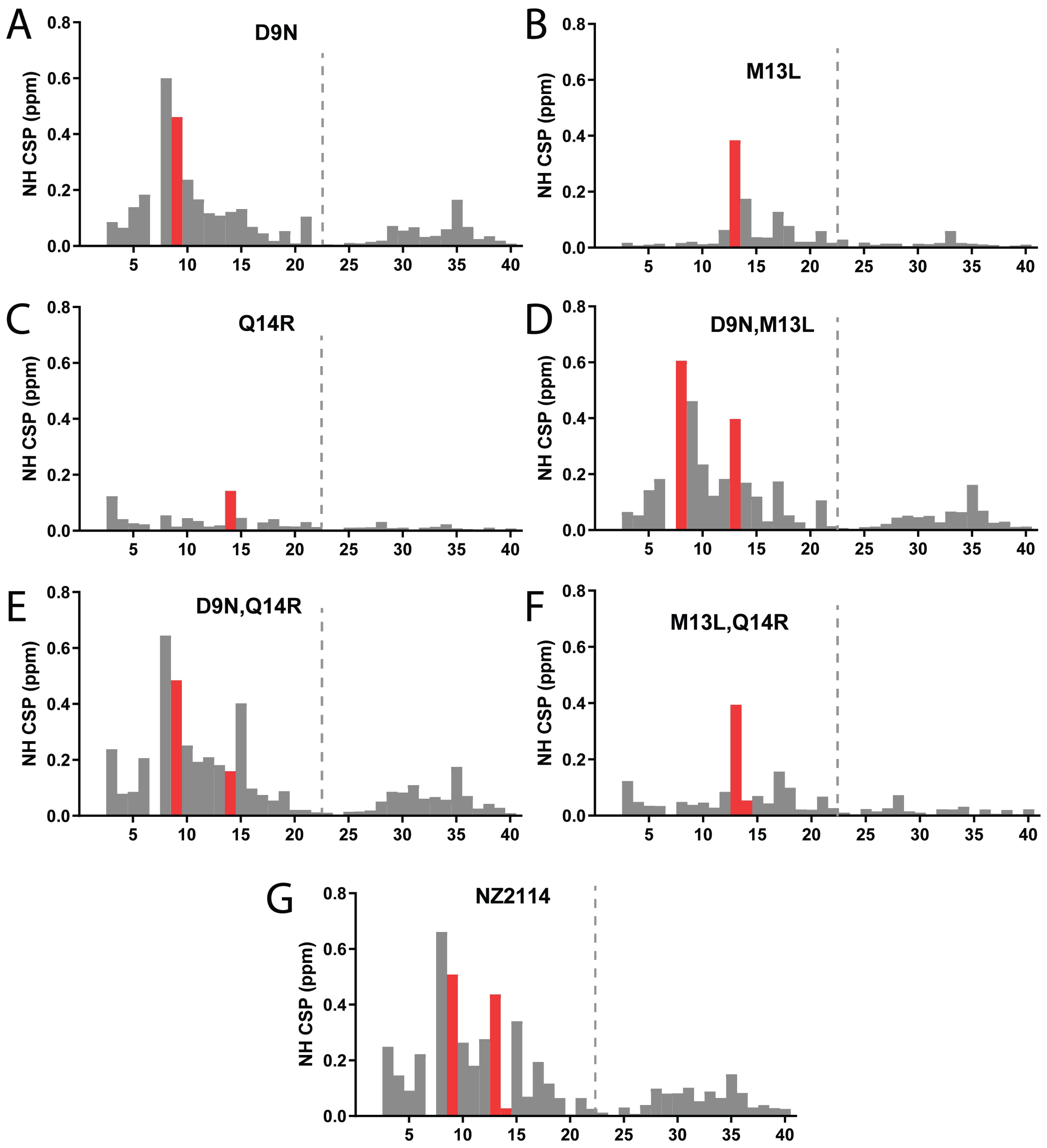

Supplementary Figure 3. Solution NMR NH CSPs of NZ2114 and intermediary mutants compared to plectasin wild-type at *low [Ca^{2+}]* conditions. Dashed line shows the cut-off used for the two average NH CPSs displayed in main text Figure 1G.

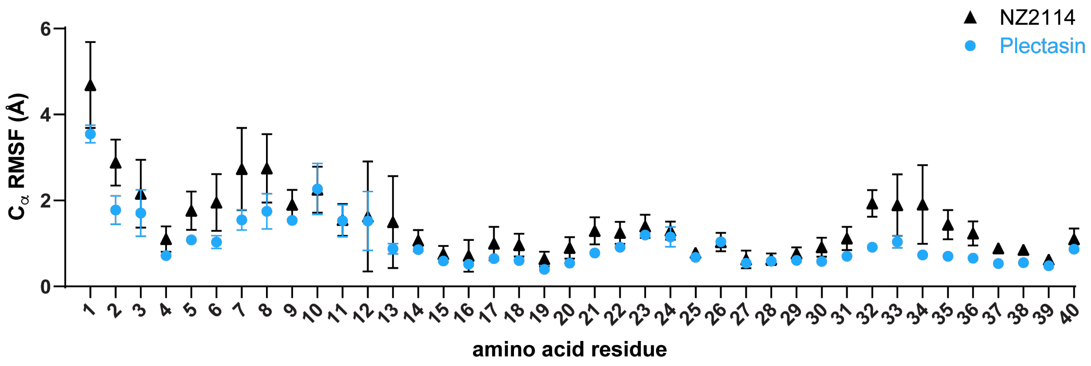

Supplementary Figure 4. MD simulations show enhanced dynamics in the N-loop of NZ2114 compared to plectasin. The graph shows the Root Mean Square Fluctuations (RMSFs) of the C_α atoms. Data show the average RMSF obtained from three replicas (100 ns of MD simulation each). The error is the standard deviation.

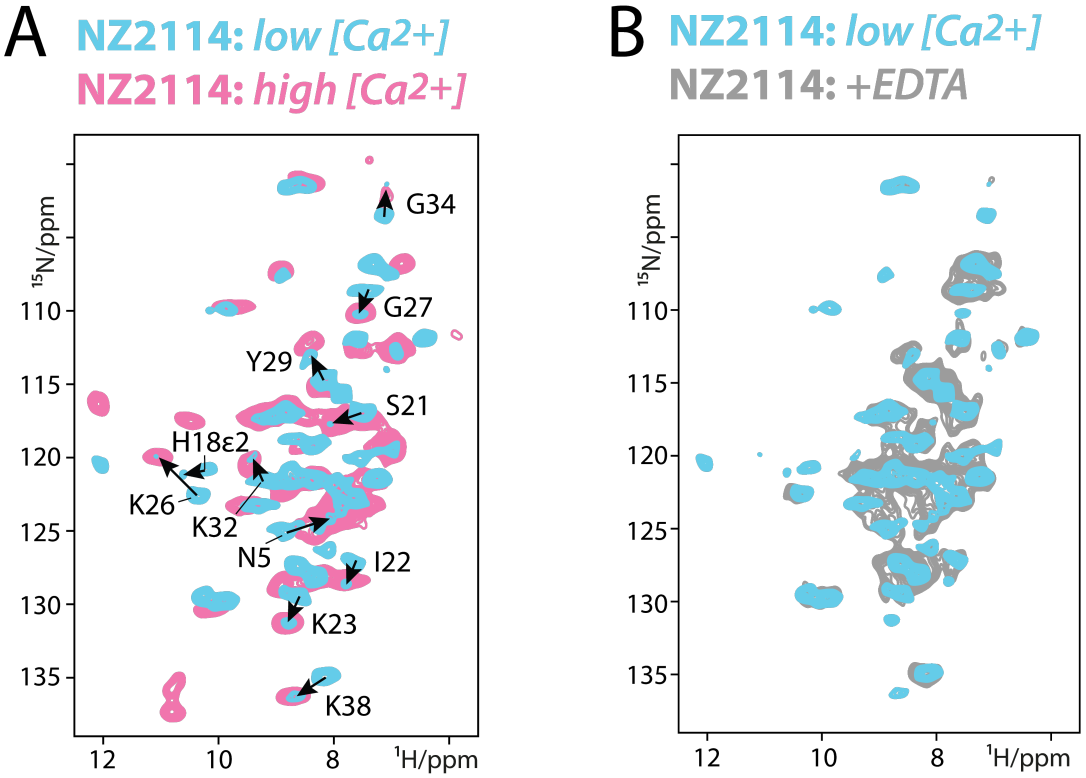

Supplementary Figure 5. (A) Superposition of proton-detected 2D NH ssNMR spectra of Lipid II-bound NZ2114 acquired at different Ca^{2+} concentration in DOPC liposomes. Examples for signals that show two different states at *low [Ca^{2+}]* are indicated by black arrows. (B) Proton-detected 2D NH ssNMR spectra of NZ2114 bound to Lipid II in liposomes without and with the addition of EDTA (blue and gray, respectively). Spectra were recorded at 16.4 T (700 MHz ^{1}H frequency), 60 kHz MAS, and at a sample temperature of 305 K.

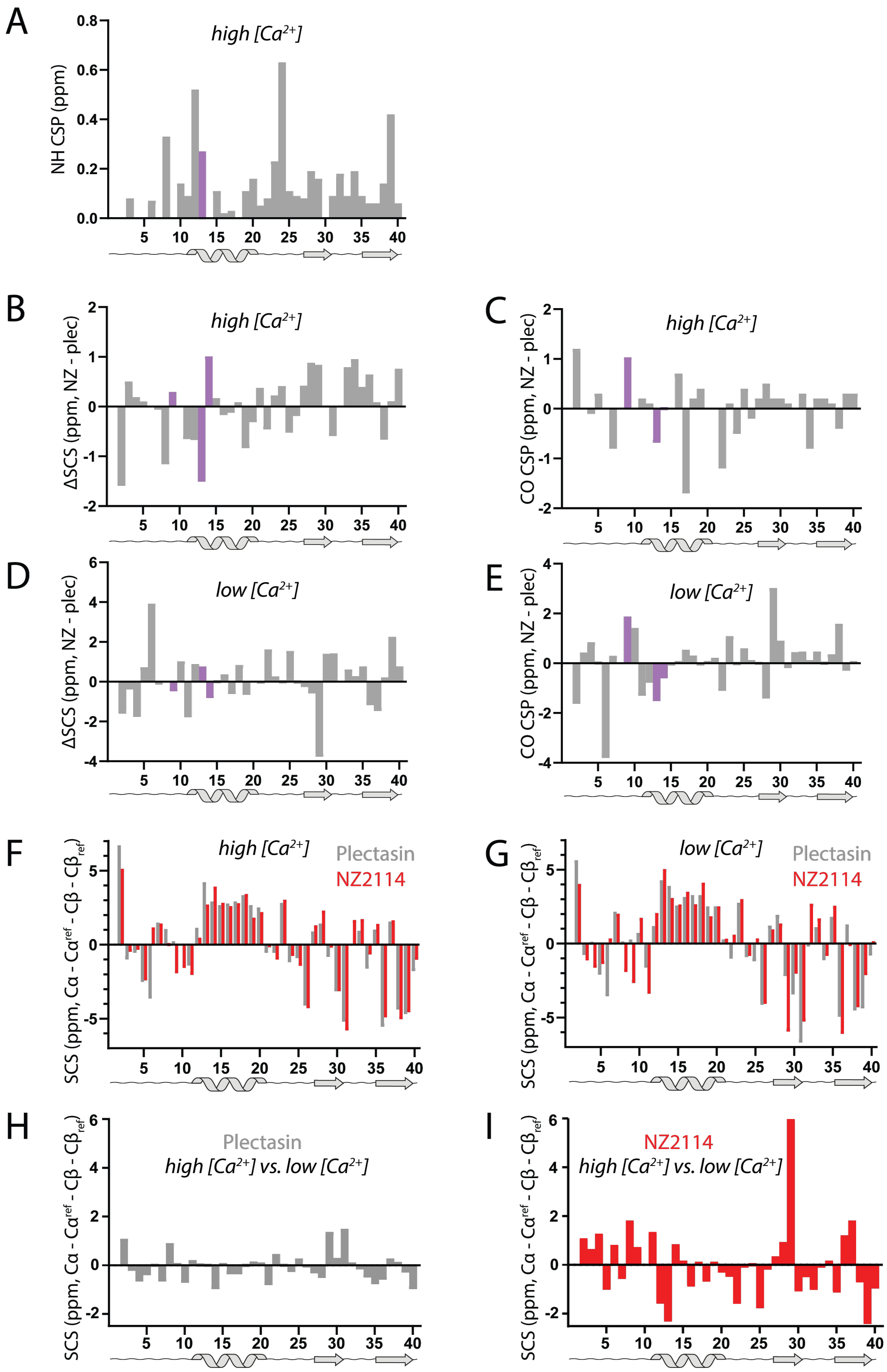

Supplementary Figure 6. (A)-(E) Quantification of ssNMR signals changes measured for plectasin or NZ2114 bound to Lipid II in DOPC membranes. CSPs and SCS changes were calculated as NZ2114 – plectasin. A,B,C) NH, SCS (CαCβ), and CO CSPs between NZ2114 and Plectasin at *high [Ca^{2+}]*. C,D) SCS (CαCβ), and CO CSPs between NZ2114 and plectasin at *low [Ca^{2+}]*. (F) and (G) show the absolute secondary chemical shifts from which (B), (D), (H) and (I) are calculated. (H) and (I) show the SCS changes for (H) plectasin and (I) NZ2114 between *high [Ca^{2+}]* and *low [Ca^{2+}] condition*. Note the much larger SCS changes for NZ2114.

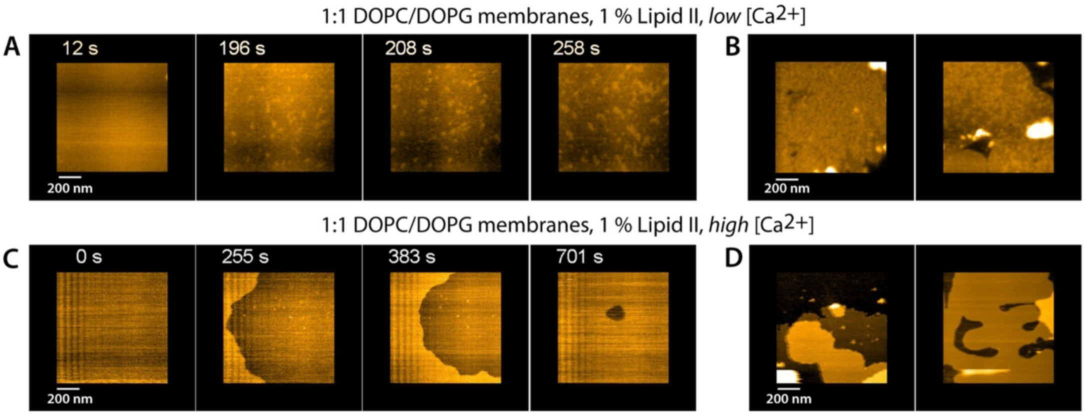

Supplementary Figure 7. (A) Snapshots of HS-AFM images following the oligomerization of NZ2114 in supported DOPC/DOPG lipid bilayers doped with 1% lipid II at *low* [Ca^{2+}]. (B) Further examples of NZ2114 oligomers, as in A, captured 15 minutes after NZ2114 application. (C) Same as A but in the presence of 1 mM Ca^{2+} (*high* [Ca^{2+}]). (D) Further examples of NZ2114 oligomers, as in C, captured 15 minutes after NZ2114 application.

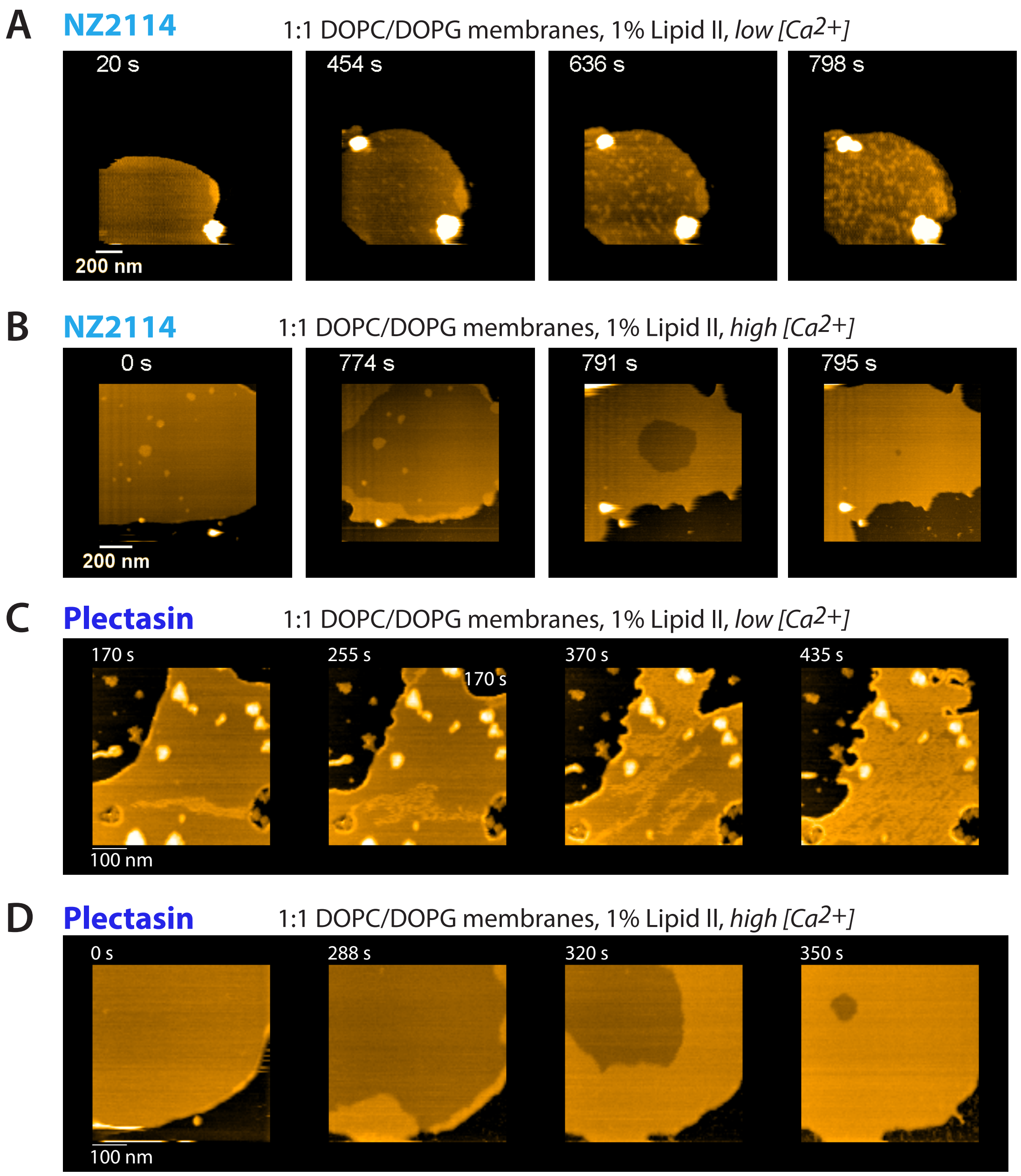

Supplementary Figure 8. (A) Snapshots of a time-lapse HS-AFM video (Supplementary Video 1) following the oligomerization of NZ2114 in supported DOPC/DOPG lipid bilayers doped with 1% lipid II. (B) Same as A but in the presence of 1 mM Ca^{2+} (Supplementary Video 2). (C) Snapshots of a time-lapse HS-AFM video following the oligomerization of plectasin in supported DOPC/DOPG lipid bilayers doped with 1% lipid II. (D) Same as C but in the presence of 1 mM Ca^{2+}. Sub-figures C and D were reproduced from Jekhmane, Derks et al. (ref. [3]).

The data show that plectasin forms a large filamentous and disordered superstructure at *low [Ca^{2+}]*, and a large supramolecular carpet at *high [Ca^{2+}]*. On the other hand, NZ2114 forms small dynamic oligomers at *low [Ca^{2+}]*, and a large supramolecular carpet at *high [Ca^{2+}]*.

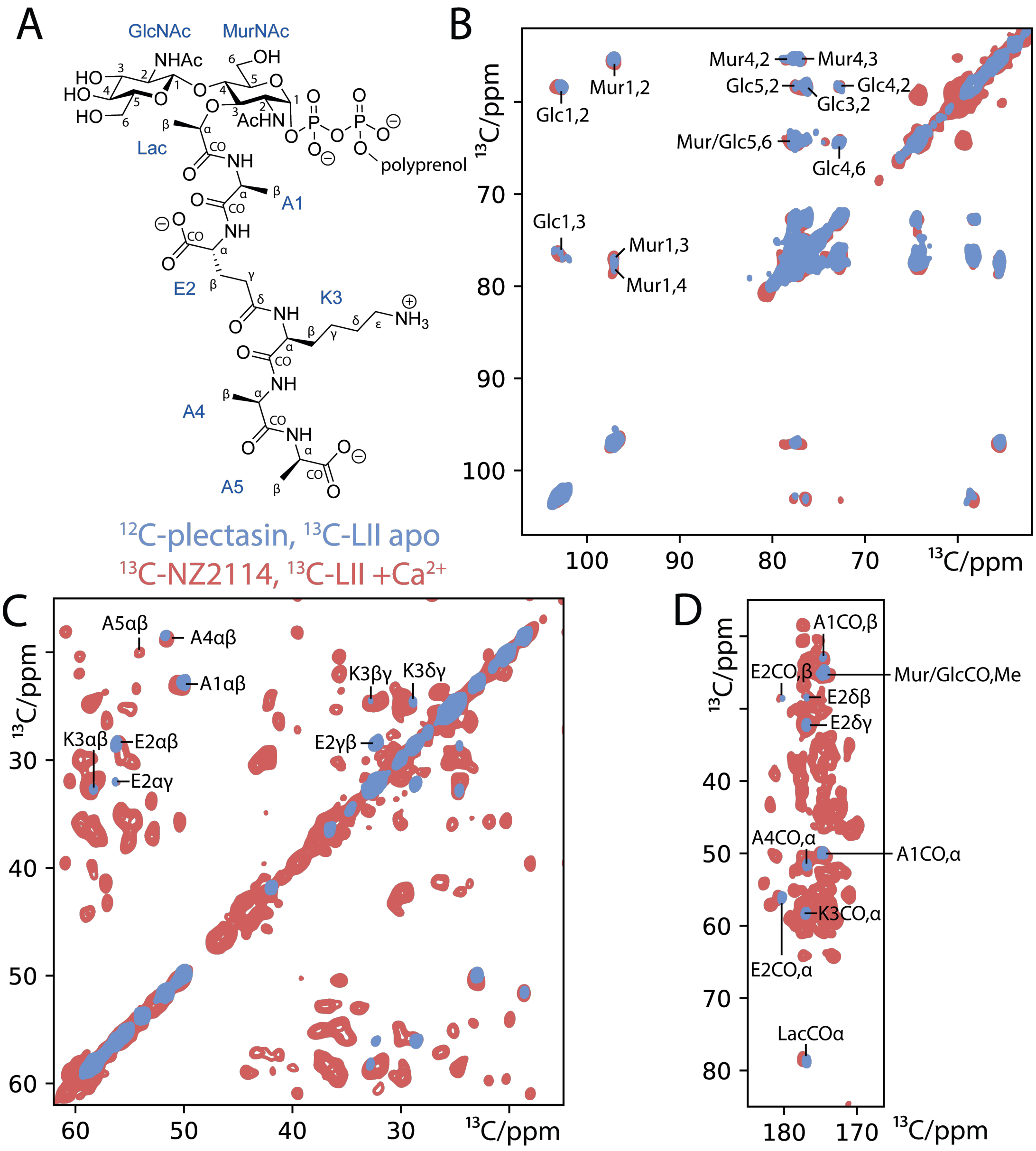

Supplementary Figure 9. Comparison of the Lipid II interface of plectasin and NZ2114. (A) Lipid II structure and atom nomenclature. (B), (C), (D) Overlays of ssNMR 2D PARIS $^{13}C^{13}C$ spectra of samples containing ^{13}C labeled Lipid II. Blue: ^{12}C-plectasin bound to ^{13}C-Lipid II in DOPC without additional 1 mM Ca^{2+}. Spectrum was recorded at 16.4 T (700 MHz ^{1}H frequency), with 42 kHz MAS, 200 ms PARIS mixing at a sample temperature of 280 K. Red: ^{13}C-NZ2114 bound to ^{13}C-Lipid II in DOPC with additional 1 mM Ca^{2+}. Spectrum was recorded at 16.4 T (700 MHz ^{1}H frequency), 15.25 kHz MAS, 50 ms PARIS mixing at a sample temperature of 270 K. (B), (C), (D) show the sugar, aliphatic and carbonyl regions respectively. Only small to moderate signal changes are observed between Lipid II bound to plectasin or NZ2114 (and upon addition of Ca^{2+}). Note that the resonance of A5αβ is not observed for plectasin due to increased dynamics of this mobile residue at the higher sample temperature. Furthermore, the subtle chemical shift changes are also due to the difference in temperature, as better agreement is observed for CC spectra recorded on ^{13}C-plectasin-^{13}C-Lipid II complex at 270 K (data not shown).

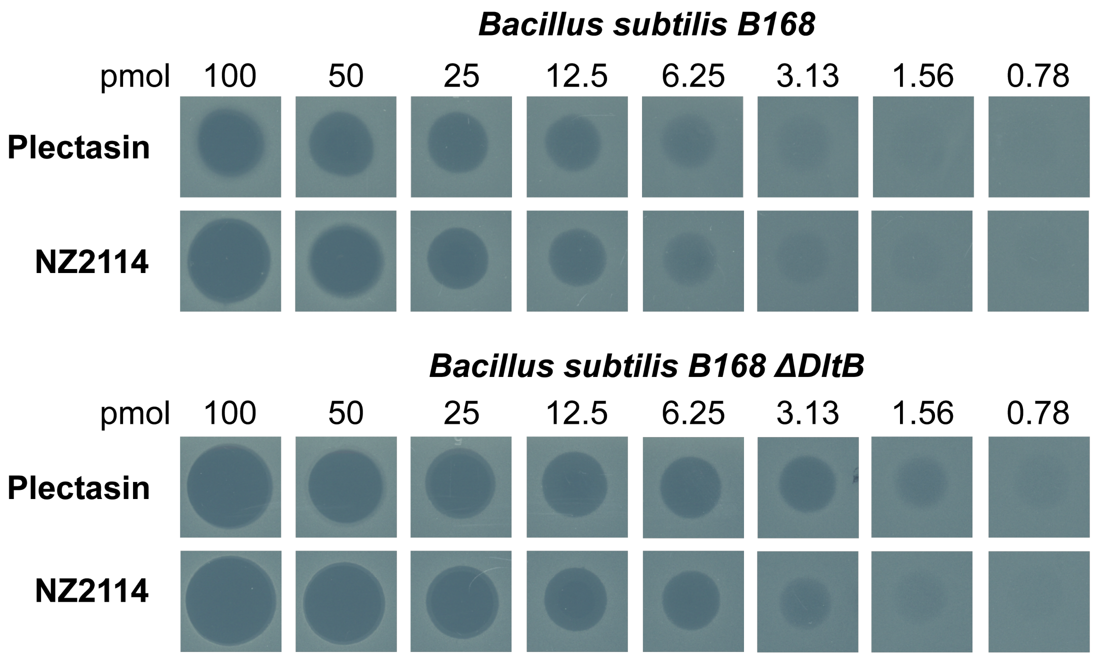

Supplementary Figure 10. Antimicrobial susceptibility assays on *B. subtilis* DltB deletion mutant. DltB is a membrane protein solely responsible for transporting D-Ala across the plasma membrane and is thereby essential for D-alanylation of teichoic acids[1,2]. This knockout strain therefore does not have any alanylation of its teichoic acids, resulting in a much more negatively charged cell envelope. Both NZ2114 and plectasin displayed a similarly moderately increased activity against the knockout strain, indicating that the cell envelope charge is not decisive for their differential activity against *Staphylococci*, despite the more positive charge of NZ2114 (+2) compared to plectasin.

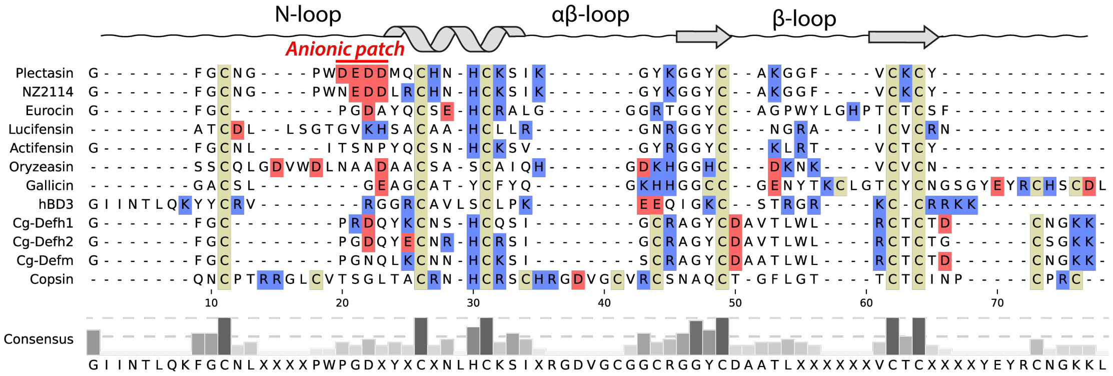

Supplementary Figure 11. Sequence alignment of defensins that have been experimentally shown to interact with Lipid II.

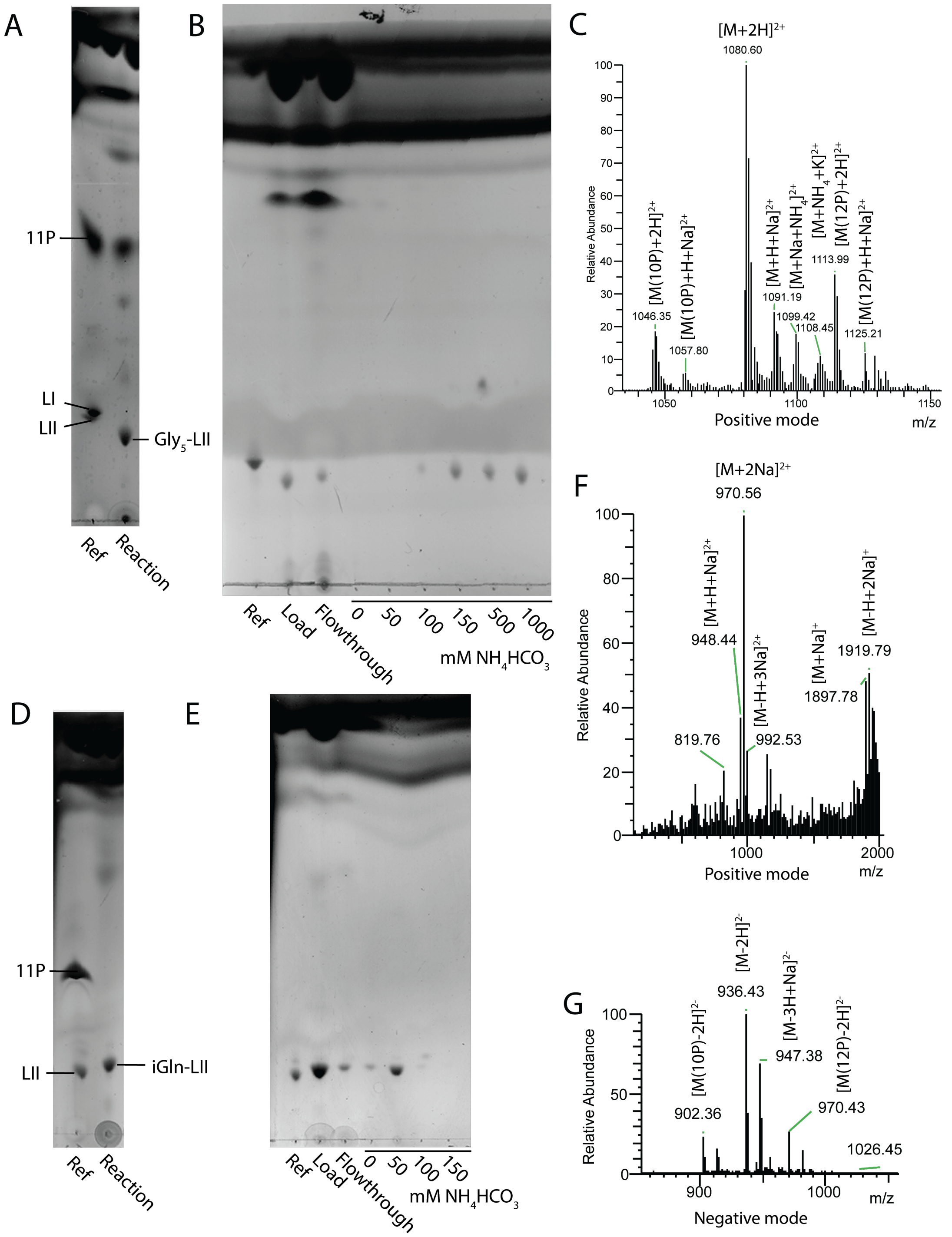

Supplementary Figure 12. Lipid II variant synthesis and purification. (A) TLC analysis of Gly5-LII synthesis reaction. (B) Purification on DEAE cellulose. (C) MS spectrum of purified Gly5-LII, displaying the characteristic distribution of polyprenols of our *Laurus nobilis* prenyl phosphate preparation. (D) Synthesis reaction of iGln-LII starting from Lipid II. (E) Purification of iGln-LII on DEAE cellulose. Note that amidated Lipid II elutes earlier compared to non-amidated Lipid II due to its decreased negative charge. (F) and (G) MS spectra of purified iGln-LII.

Supplementary Table 1

MIC assays of plectasin, NZ2114, and intermediary mutants (in µg/mL)

	***S. aureus* USA300 (MRSA)**	***S. aureus* ATCC29213 (MSSA)**	***S. simulans* 22**
Plectasin	12.5	12.5	1.56
D9N	6.25	12.5	0.78
M13L	25	25	3.13
Q14R	6.25	6.25	≤ 0.78
D9N,M13L	6.25	12.5	0.78
D9N,Q14R	3.13	3.13	≤ 0.39
M13L,Q14R	6.25	6.25	≤ 0.78
NZ2114	3.13	3.13	≤ 0.78
Vancomycin	1.56	1.56	0.78-1.56

Supplementary Table 2:

Solution NMR chemical shift assignments of NZ2114

Residue	H^N	N	Cα	Cβ	C
**G1**					
**F2**					
**G3**	8,09	101,1	44,69	-	174,33
**C4**	7,64	121,9	53,01	34,49	173,78
**N5**	8,08	121,2	52,19	39,46	174,11
**G6**	7,39	109,6	43,27	-	
**P7**	-	-	63,8	30,67	177,28
**W8**	7,9	117,9	57,35	27,45	175,64
**N9**	7,98	121	53	38,81	172,91
**E10**	8,4	121,3	56,35	30,08	175,2
**D11**	8,19	124,3	52,43	40,49	175,09
**D12**	8,17	122,2	57,23	41,21	177,72
**L13**	8,07	120	57,35	41,07	178,49
**R14**	7,61	120,5	59,34	29,56	179,25
**C15**	7,54	120,6	58,38	35,39	175,42
**H16**	8,94	122,2	60,75	31,26	177,39
**N17**	8,57	115	55,77	37,41	178,38
**H18**	8,01	122,1	59,57	28,77	178,05
**C19**	8,62	120,2	58,41	36,68	175,86
**K20**	7,78	114,2	58,24	31,11	176,84
**S21**	7,34	114,4	59,34	63,65	173,23
**I22**	7,55	125,9	60,74	37,72	175,31
**K23**	8,11	126,4	58,28	31,43	177,06
**G24**	8,45	112,9	44,76	-	174,44
**Y25**	7,68	119,6	56,6	38,37	175,31
**K26**	10,61	120,7	55,82	34,34	176,3
**G27**	7,27	106,9	45,62	-	169,84
**G28**	8,77	107,6	47,02	-	170,72
**Y29**	8,42	115,5	55,93	39,89	173,12
**C30**	9,05	117,6	53,24	36,36	174,88
**A31**	9,49	129,3	51,01	21,88	176,73
**K32**	8,88	119,3	56,72	29,8	174,22
**G33**	8,75	111,2	45,74	-	174,22
**G34**	7,43	104,2	44,76	-	174,22
**F35**	7,34	116,9	60,04	40,93	175,31
**V36**	7,7	118,4	60,74	34,99	174,22
**C37**	8,75	127,2	55,5	35,04	172,25
**K38**	8,99	135,9	54,53	33,59	173,45
**C39**	7,83	121,1	51,13	34,01	174,33
**Y40**	8,31	126,2	58,28	39,76	

Supplementary Table 3:

ssNMR chemical shift assignments of NZ2114 bound to Lipid II in DOPC membranes at *high* *[Ca^{2+}]*.

Residue	H^N	N	Cα	Cβ	C
**G1**					
**F2**			59,87	37,23	174
**G3**	8,44	101,42	44,85	-	175,3
**C4**	8,36	125,04	53,62	34,67	173,8
**N5**	8,08	123,76	52,85	40,54	173,2
**G6**	6,82	106,87	46,51	-	170,4
**P7**	-	-	64,68	31,59	175,9
**W8**	5,86	111,8	56,1	27,85	177,5
**N9**	8,27	124,82	52,83	40,04	172,8
**E10**	8,48	124,64	55,79	30,99	175
**D11**	8,26	128,37	50,77	39,49	175,4
**D12**	6,98	119,67	57,7	43,9	178,1
**L13**	8,46	119,43	57,67	41,98	177,9
**R14**	7,93	123,79	59,8	30,44	178,5
**C15**	7,3	121,57	59,02	36,89	175,6
**H16**	9,61	123,28	61,27	32,5	177,7
**N17**	9,01	116,93	55,69	38,17	175,9
**H18**	8,02	122,73	60,26	30,67	178,8
**C19**	8,85	122,19	58,75	37,62	176
**K20**	8,22	115,27	57,89	31,86	176,5
**S21**	7,49	117,49	59,39	65,09	173,4
**I22**	7,81	128,49	61,35	39,98	176,4
**K23**	8,81	131,27	59,37	32,5	176,7
**G24**	8,82	116,33	44,57	-	173,2
**Y25**	8,37	121,35	59,03	41,45	175,3
**K26**	11,06	120,03	55,5	35,96	174,4
**G27**	7,56	110,32	46,65	-	170,5
**G28**	9,75	109,86	47,64	-	171,6
**Y29**	8,41	112,22	56,95	37,92	173,8
**C30**	9,25	117,52	53,37	37,21	175,1
**A31**	10,81	137,26	51,26	23,41	176,9
**K32**	9,49	120,49	56,48	30,99	176,1
**G33**	8,95	107,43	47,07	-	172,6
**G34**	7,11	102,08	44,68	-	174
**F35**	7,33	119,18	61,52	42,6	175,1
**V36**	7,95	117,6	60,3	36,08	176,1
**C37**	10,11	130,35	56,36	35,41	171,5
**K38**	8,7	136,25	55,36	36,56	174
**C39**	7,88	123,43	50,97	36,24	174,9
**Y40**	8,91	128,83	59,1	41,11	178,3

Supplementary Video 1: HS-AFM movie showing the dynamic interaction of NZ2114 with SLB composed of DOPC, DOPG and L II in the absence of Ca^{2+}. Image acquisition rate is 1 frame per second.

Supplementary Video 2: HS-AFM movie showing the dynamic interaction of NZ2114 with SLB composed of DOPC, DOPG and L II in the presence of 1 mM Ca^{2+}. Image acquisition rate is 0.5 frames per second.

References

1 Hyyryläinen, H.-L. *et al.* d-Alanine Substitution of Teichoic Acids as a Modulator of Protein Folding and Stability at the Cytoplasmic Membrane/Cell Wall Interface of Bacillus subtilis. *Journal of Biological Chemistry* **275**, 26696–26703 (2000). https://doi.org/10.1016/s0021-9258(19)61432-8

2 Zhang, P. & Liu, Z. Structural insights into the transporting and catalyzing mechanism of DltB in LTA D-alanylation. *Nature Communications* **15** (2024). https://doi.org/10.1038/s41467-024-47783-7

3 Jekhmane, S. *et al.* Host defence peptide plectasin targets bacterial cell wall precursor lipid II by a calcium-sensitive supramolecular mechanism. *Nature Microbiology* **9**, 1778–1791 (2024). https://doi.org/10.1038/s41564-024-01696-9
